# Microprocessors in pathology

**DOI:** 10.1155/S1463924684000092

**Published:** 1984

**Authors:** F. Murphy

**Affiliations:** Pathology Department Mount Vernon Hospital Northwood Middlesex HA6 2RN UK


Journal of Automatic Chemistry, Volume 6, Number 1 (January–March 1984), pages 44–45

### Microprocessors in pathology*

**F. Murphy**

*Pathology Department, Mount Vernon Hospital, Northwood, Middlesex HA6 2RN, UK*

Articles on the use of computer systems, both mainframe and minicomputer, in pathology laboratories have been prolific over the last few years. It might therefore be assumed that a discussion on the role of the microprocessor is essentially similar but on a smaller and humbler scale. I hope to show, however, that the microprocessor has a very distinct and significant role in the pathology department—it is increasingly becoming an indispensable component or 'tool' in analytical methodology and instrumentation.

The essential factor in distinguishing the microprocessor or microcomputer (in simple terms a microprocessor plus an operating system, input and output facilities and user memory) from the mini- or mainframe computer is its relatively low cost. The price of a fully comprehensive computer system for many medium and small-sized laboratories is prohibitive, but the 'micro' has changed this cost perspective and puts into the hands of the laboratory worker, or gives to an individual analytical procedure, tremendous computing power than can truly be said to be personal. The computer is under the control of and can be developed by the analyst him/herself for specifically tailored situations and needs that can develop commensurately with changes in the wider laboratory environment. It also means that much of the analytical computation, data handling and formatting, quality control, and the generation of hard-copy results and work-lists can be performed off-line by a local work-station. This can improve the turnover time for obtaining results.

In many applications the microprocessor is 'embedded' within an instrument so that it controls certain features of the instrument but is 'invisible' to the user. From current laboratory magazines it would appear that for an instrument just to have a microprocessor gives it a distinct selling advantage. A summary of some uses that micros are being put to in laboratory practice include the following.

### Control of mechanical processes (automation)

These may be pre-programmed, when a fixed sequence of events is followed, or may involve reactive processes in which some form of feedback loop is involved. For this sensors and transducers monitor and modify the environment via output/input ports. A simple example is the micro-controlled histology tissue processor in which tissue held in a rack is sequentially immersed in baths of fluid at controlled temperatures for varying times. Another example of mechanical control is the programmable X-Y sampler. Test, standard and control solutions from a variety of racks can be sampled and dispensed, in a different order into an arrangement of racks or trays, based on the X-Y co-ordinates of the platform. This can be very useful in automating sample presentation.

### Computation of data

(1) *Arithmetic manipulation.* In pathology many values are derived from other measured values. For example in haematology using the measured values of haemoglobin concentration, red blood cell count and haematocrit percentage the derived values of mean cell volume [MCV], mean cell haemoglobin [MCH] and mean cell haemoglobin concentration [MCHC] can be obtained.

(2) *Logical computation.* The microprocessor is able to make logical decisions on the result of analytical data about a preferred further course of action. This can be of the form: 'If A is true, take path B, otherwise take path C'. Complex examples are to be found in the area of diagnosis.

(3) *Statistical computation.* This is the computation of results to indicate quantitatively the accuracy and precision of the data. With the concept of the 'expected' or 'normal' range it is essential to know what degree of confidence can be placed upon a result, or upon differences in results. With the local microprocessor, within-batch and between-batch statistical analysis is readily obtainable, so that unacceptability of results can be recognized and dealt with speedily and efficiently.

(4) *Interpretive computation.* In the light of results for a test, or set of tests, one determines which further tests will assist in deciding on a more definite diagnosis. For such computation results may need varying weightings. With multi-analysers, results are often available that have not been requested. The profile of results from the requested tests may indicate the value of reporting other results also. Patient age, sex, clinical details etc., will also influence such decisions.

(5) *Extrapolative/predictive computation.* It is often necessary to derive test results by extrapolation from results obtained with a finite number of discrete standard solutions. In simple form this may be the construction of a standard curve from two or more known points, to the more complex computation involved in enzyme kinetic and radioimmunoassay techniques.

### Self-calibration and feedback control mechanisms

The microprocessor, for example, may automatically detect and control drift which may be due to such factors as change in ambient temperature, saturation of membranes etc.

### Auto-diagnostic routines

The microprocessor can self-test its own circuits and instrument features. This is useful in trouble-shooting and in fault-identification, especially with the increasing complexity of modern instrumentation.

### Predictive or warning systems

This is related to the two above in which the microprocessor monitors the system status and gives warning when certain control features approach predetermined limits of tolerance. Thus indication is given that, although the instrument is operating within acceptable limits, it requires attention before a fault develops.

*This paper was first published in *Journal of Medical Engineering & Technology (1983).*

### Data storage for analytical work

The microprocessor can direct the storage of data or results when, for example, the result is one of a series, perhaps over a 24 h period. Examples of this are water deprivation tests, absorption tests etc.

### Data management

An example of this would be the ability to collate results. Randomly ordered analyses on a sequential analyser can be sorted into a variety of groups: in laboratory number order, ward list order etc.

### Personal experiences

Within our own laboratory we have implemented the following uses of microcomputer systems.

### *Gilford 203 system clinical analyser and the BBC microcomputer*

We have linked the Gilford to the BBC micro via its RS 423 interface. The BBC has its own local high-speed printer and also links up to the main laboratory computer, again through the RS 423. This gives the following additional facilities at the local level:

(1) The BBC can further process results, i.e. determine globulin concentration from the difference between total protein and albumin concentrations.
(2) Further results and details can be added to the results slots, for instance coded electrophoretic patterns, comments from analyst or other manually derived results.
(3) Patient identification, sample history and other investigations requested may be obtained locally by direct interrogation of the main computer.
(4) Quality control can be assessed at the local work-station and unacceptable or questionable results can be investigated immediately.
(5) All data, results, comments etc. can be dumped directly into the mainframe computer, rapidly and in any format.
(6) Local print-outs can be produced. They may be used for urgently required results and also as a back-up against main computer failure and other down times.

The feasibility of controlling the spectrophotometer and sampler/dispenser components directly using a Hewlett-Packard desk-top microcomputer has also been investigated and found to be quite possible.

### *Gamma counter–Hewlett-Packard link*

The system configuration is a Nuclear Enterprise 1600 gamma counter linked to a Hewlett-Packard programmable microcomputer via an RS 232C interface. The micro performs data reduction of radioimmunoassays such as thyroxine, cortisol, TSH etc. Different radioimmunoassay tests yield different theoretical best-fit curves of known ligand concentration plotted against a function of the gamma-emmission count rate. The micro assesses the results for the best fit of four curve-fit methods: linear, semi-log, log-logit, and non-linear curve fits. The microcomputer also produces replicate spread percentage fitting error and goodness-of-fit values for each curve and will give a curve print-out for visual inspection. In these curve-fit methods the points may have to be weighted. The spline approximation method will connect all points with equal weighting. Spline plotting requires large memory capacity which has previously limited its use. However, modern micros with increased memory capacities have overcome this.

### *LKB–PET link*

Both the LKB calculating absorptiometer and the LKB reaction rate analyser have been linked to a Commodore PET microcomputer. The spectrophotometer has an automatic feed mechanism to handle up to 100 tubes for end-point chemistries: output from the LKB is in the form of BCD (Binary Coded Decimal) and so is put through a BCD–digital interface for the serial IEEE interface port of the PET.

The PET can monitor the enzyme reaction rates for linearity, and, if linearity is achieved within pre-determined time and limits, the PET can trigger the mechanism to add a starter reagent to the next test assay and move it into the read position.

### *Technicon SMA II–BBC microcomputer link*

The SMA's (Sequential Multiple Analyser) own microprocessor is primarily for function monitoring. This includes monitoring of dwell time and curve evaluation with peak error detection. The SMA is linked to the main laboratory computer via a BBC microcomputer. Although the SMA has an RS 232 interface, once data transfer has commenced it is not possible to interrupt without causing a system error condition. This lack of handshaking means data transfer must occur at a slower rate than might otherwise be possible. It is to be hoped that manufacturers will recognize the growing requirements for laboratories to interface instruments with a variety of microprocessors and so offer true handshaking facilities. Interfaces such as the IEEE and RS 232 should be implemented in standard ways.

The BBC's role is to allow local editing before all results are dumped into the main computer. This involves collation of results to produce different test profile groups and the addition of other results obtained off-line, i.e. osmolalities, and the print-out of local result sheets. The switching of the BBC from the SMA to the main computer is achieved automatically by an electronic switch which is controlled by the BBC parallel user port.

### Conclusions

'Distributive' processing based upon several microprocessors each dedicated to a specific laboratory section, networked and sharing a common large data-base with multi-user capabilities may be an alternative to a single, central, large computer in the laboratory. This provides maximum flexibility of the system. Units can be interchanged and the system can be updated at a pace and cost suited to the particular laboratory's resources. Modification and development can be easily achieved without disrupting the whole system. The computer becomes a piece of laboratory equipment and not the sole province ot the 'computer professional'.

The attraction of the microprocessor to the laboratory is its extreme adaptability which brings growing computing power, at a comparatively low cost, into the hands of the laboratory worker. Increasingly, the use of such microprocessing capability is limited only by the imagination of the user.

